# Quality of Communication Between Professors and University Students in the Process of Learning

**DOI:** 10.1590/0103-6440202406081

**Published:** 2024-10-28

**Authors:** Carlos Estrela, Marcela Gimenes B Oshita, Matheus F Perazzo, Ana Helena G Alencar, Júlio A Silva, Lucas RA Estrela, Luciano TA Cintra, Cyntia RA Estrela

**Affiliations:** 1 Professor of Department of Stomatologic Sciences, School of Dentistry, Federal University of Goiás, GO, Brazil;; 2 Professor of post-graduate course, People Management Course (PECEGE Institute), University of São Paulo-Esalq, SP, Brazil;; 3 Department of Preventive and Restorative Dentistry, School of Dentistry, São Paulo State University (UNESP), SP, Brazil;; 4 Department Oral Biology, School of Dentistry, Evangelical University of Goiás, GO, Brazil

**Keywords:** Learning, communication, teaching, dental education, Dentistry

## Abstract

Teaching requires the incorporation of communication skills, and these potentials may affect the outcomes of the learning process. This study evaluated the quality of communication between professors and students during the process of learning Dentistry. A questionnaire was developed and applied to evaluate their perceptions about their communications in an undergraduate Dentistry course. The questionnaire had ten items to analyze professors’ self-perception and ten for students’ perceptions, using a Likert-like scale and a final open question. During its construction, it was sent to five specialists to analyze content validity. The open question gathered suggestions to improve and intensify communications effectively and to identify vulnerabilities. Total scores ranged from 10 to 50, with 10 as the worst perception, and 50, as the best. The scores were calculated by adding all answers and then dividing that sum by the total number of items. Cronbach’s alpha was calculated to evaluate the instrument’s internal consistency. The level of significance was set at 0.05%. The Student *t*-test was used to determine differences between groups. Professors’ self-perceptions and students' perceptions of the quality of communication of the university teaching staff during the student's learning process had statistically significant differences. Professors classified their ability to communicate when emitting and receiving information as satisfactory. Students, however, did not fully agree with them, particularly on the items about receiving information. The perceptions identified in this study may lead to a new direction in the communicative behavior of professors and students.

## Resumo

O ensino da odontologia requer a incorporação de habilidades de comunicação, e essas qualidades podem afetar os resultados do processo de aprendizagem. Este estudo avaliou a qualidade da comunicação entre professores e alunos durante o processo de aprendizagem da Odontologia. Foi desenvolvido e aplicado um questionário para avaliar suas percepções sobre suas comunicações em um curso de graduação em Odontologia. O questionário continha dez itens para analisar a autopercepção dos professores e dez para a percepção dos alunos, utilizando uma escala do tipo Likert e uma questão final aberta. Durante sua construção, foi enviado a cinco especialistas para análise de validade de conteúdo. A pergunta aberta reuniu sugestões para melhorar e intensificar a comunicação de forma eficaz e identificar vulnerabilidades. A pontuação total variou de 10 a 50, sendo 10 a pior percepção e 50 a melhor. As pontuações foram calculadas somando todas as respostas e dividindo essa soma pelo número total de itens. O alfa de Cronbach foi calculado para avaliar a consistência interna do instrumento. O nível de significância foi fixado em 0,05%. O teste t de Student foi utilizado para verificar diferenças entre os grupos. A autopercepção dos professores e a percepção dos alunos sobre a qualidade da comunicação do corpo docente universitário durante o processo de aprendizagem dos alunos apresentaram diferenças estatisticamente significativas. Os professores classificaram como satisfatória a capacidade de comunicação ao emitir e receber informações. Os alunos, porém, não concordaram plenamente com eles, principalmente nos itens sobre recebimento de informações. As percepções na habilidade comunicação identificadas neste estudo podem levar a um novo rumo no comportamento comunicativo de professores e alunos.

## Introduction

Communications in the context of human life characterize an essential process and product that induces interactions at different social levels. Communications motivate interchanges and coexistence in the family, at work, and in social relationships, all supported by the incorporation of content and forms contained in messages between interlocutors ^1^. 

The power of human interactions has grown stronger due to innovations in the area of information technologies. The knowledge and responsibility brought by communication into people's lives and society underscore the importance of this activity in human, school, and university formation, as well as at work. Personal and professional success has been associated with excellence supported by good quality communications. At the same time, noise and negative interferences may be harmful to the assertive potential of any interlocutory action ^1^. Communication should be understood as a constructive process of human relationships. Its scope shows the responsibility to stimulate increased development of service segments and organizational structure, with important potential to change the management and structure of personal behavior. In this sense, effective communication brings benefits in all aspects of the learning process, health, and service, in addition to valuing the relationships between teachers, students, patients, employees, and managers ^2^ ^,^ ^3^ ^,^ ^4^ ^,^ ^5^ ^,^ ^6^.

Learning strategies have been the focus of in-depth discussions over time. In Bloom’s definition of the learning process (1956)^7^, students should have an active role in their practice (action), which should include knowledge, abilities, and attitudes. 

The dynamics and complexity of learning must be analyzed within a systemic view. From this perspective, communication goes beyond the power of public speaking, redirecting it toward a greater meaning, clear, logical, formative, and effective. 

Possible failures in university learning environments may originate in poor communication. Preparing professors to be competent in oral communication has been one of the needs identified during teaching courses ^8^ ^,^ ^9^ ^,^ ^10^ ^,^ ^11^. The communicative competence acquired by professors during their teaching formation has been discussed ^9^, whose findings revealed that not enough work has been assigned to the development of this ability. The level of this ability is, therefore, low. Professors’ perception of the communicative competence of their students has pointed to a serious disconnection between their initial formation and what is required for their professional performance ^10^. Bandrés et al. ^8^ evaluated the perceptions that students preparing to become professors had of their competence in verbal communication and of the education received, and explored their perceptions according to the different dimensions that compose this competence. Findings revealed a negative correlation between the evaluation of the education received and the course, whereas students about to graduate evaluated it as insufficient. 

Teaching requires the incorporation of communication skills, and these characteristics may affect the outcomes of the learning process ^8^ ^,^ ^7^ ^,^ ^8^ ^,^ ^9^ ^,^ ^10^ ^,^ ^11^ ^,^ ^12^ ^,^ ^13^ ^,^ ^14^ ^,^ ^15^ ^,^ ^16^ ^,^ ^17^ ^,^ ^18^ ^,^ ^19^ ^,^ ^20^ ^,^ ^21^ ^,^ ^22^ ^,^ ^23^ ^,^ ^24^ ^,^ ^25^ ^,^ ^26^. Professor-patient-student communications are also important factors and deserve special attention ^3^ ^,^ ^4^ ^,^ ^5^ ^,^ ^12^ ^,^ ^13^ ^,^ ^14^ ^,^ ^15^ ^,^ ^16^ ^,^ ^17^ ^,^ ^18^ ^,^ ^19^. Some competencies are part of this context, such as the need to conduct dialogues, to be a proactive listener, to keep focus and attention, and to take the lead in accurate decision-making to facilitate learning. The development of communication skills has been studied at different educational levels because of its importance and the assertiveness of positive outcomes ^3^ ^,^ ^4^ ^,^ ^5^ ^,^ ^12^ ^,^ ^13^ ^,^ ^14^ ^,^ ^15^ ^,^ ^16^ ^,^ ^17^ ^,^ ^18^ ^,^ ^19^ ^,^ ^20^ ^,^ ^21^.

Development and training in a corporative organization or university strengthens a learning culture in which the education of its members is one of the basic pillars of professional formation and careers ^22^. Therefore, in addition to technical skills, behavioral skills are part of the communicative process, and several other factors should be taken into consideration, such as the abilities to keep dialogues, avoid conflict, resolve differences, eliminate noise, be creative, collaborative, flexible, resilient, proactive and emotionally intelligent and exercise leadership ^22^. 

In the current context of professional formation in Dentistry, the relationship between professors and students, as well as between professionals, patients, and students, demands competence and communicative skills ^1^ ^,^ ^2^ ^,^ ^3^ ^,^ ^4^ ^,^ ^5^ ^,^ ^7^ ^,^ ^8^ ^,^ ^9^ ^,^ ^10^ ^,^ ^11^. There is room for improvement in the process of learning Dentistry, as new skills should be incorporated to improve the quality of communications. An adequate performance as a facilitator requires pointing out the importance of students having control of the systemic dynamics and the leadership in the learning process. 

As there are few studies about knowledge of verbal communication in Dentistry, this study utilized a questionnaire to evaluate communications between professors and students in the process of learning Dentistry. Thus, this study evaluated the self-reported perceptions of the ability to communicate among professors and students during the process of learning Dentistry.

## Material and Methods

This study used information obtained from the responses to a questionnaire applied to professors and students in the third to fifth year of the undergraduate course in Dentistry at a Higher Education Institution. This study was approved by the Ethics in Research Committee of the institution where it was conducted (#66175722.5.0000.5083).

Professors included were regular university employees no longer on probation. All teachers selected for the study are aware of the different teaching modalities. All teachers are invited to participate in a teaching training course offered by the University before becoming a teacher. The students had completed at least two years of Dental School, as this would ensure that they had taken more courses in specific formative areas. From this moment on, students have already come into contact with the contents of scientific methodology. Data were collected from January to March 2023. 

Selected professors and students read and signed an informed consent term containing the study objectives. Their participation was voluntary, free, and conscious, and the data collected were used only for research. The privacy and confidentiality of their responses were assured, and their consent could be withdrawn at any time.

The study model was based on a questionnaire about perceptions of communicative competence between professors and students in an undergraduate course of Dentistry at a Higher Education Institution. 

The initial questionnaire version was sent to five specialists in the construct under analysis for the analysis of content validity (Table 1 and 2). The specialists received a chart to evaluate the questionnaire. If they thought that any item had to be changed to better describe the behavior under study, they filled out a specific box in the chart with their suggestion on how that item should be written. At the end of it, there was an area to indicate which items were thought to be redundant.

The questionnaire was separated into two parts, both with 10 items each. One was for the analysis of professors’ self-perceptions of emission and reception, and the other was for the analysis of students’ perceptions. Each had a five-point Likert scale ^23^ and an open question at the end. The questionnaires evaluated professors’ self-perception of communicative competence as senders and receivers of information (10 items) (Table 1), and students’ perception of the quality of their professors’ verbal communicative skills (10 items) (Table 2). Both groups, professors, and students, had an open question about communication skills in the process of learning Dentistry. The closed questions were statements about which the participants had to give their opinions. The answers had five options, from the least to the most favorable. In questions about frequency, the options were 1 - never; 2 - rarely; 3 - sometimes; 4 - often; and 5 - always, and in questions about agreement, 1 - strongly disagree; 2 - disagree; 3 - neither agree nor disagree; 4 - agree; 5 - strongly agree. The open questions asked participants to make suggestions to improve and make communications more effective and to identify vulnerabilities and the most important difficulties in communications, those that might have an impact on the process of learning Dentistry. The total score ranged from 10 to 50, where 10 indicated the worst perception of communications quality, and 50, was the best. The general questionnaire scores were calculated by adding the answers to all items and then dividing that sum by the total number of items. Cronbach's alpha (α) was calculated to evaluate the internal consistency of the instruments used in the study.

The answers were tabulated and analyzed statistically using the Statistical Package for Social Sciences (IBM SPSS Statistics para Windows 25.0, IBM Corp., Armonk, NY). The level of significance was set at 0.05. The Student *t*-test was used to determine differences in answers between groups.

**Table 1:** Questionnaire about professors’ self-perceptions of the quality of communications with students in the process of learning Dentistry.

Professors’ self-perception (Competence as sender/receiver)	Never 1	Rarely 2	Sometimes 3	Often 4	Always 5
1. I develop appropriate teaching methods for different learning situations.	0(0.0%)	0(0.0%)	12(26.1%)	27(58.7%)	7(15.2%)
2. I present content in a logic and coherent context.	0(0.0%)	0(0.0%)	0(0.0%)	27(58.7%)	19(41.3%)
3. I have communications skills and make clear analogies.	0(0.0%)	0(0.0%)	4(8.7%)	25(54.3%)	17(37%)
4. I use and competently apply new technologies as strategies in the learning process.	0(0.0%)	5(10.9%)	16(34.8%)	20(43.5%)	5(10.9%)
5. I am confident about the quality and impact of my communications on students’ learning.	0(0.0%)	0(0.0%)	12(26.1%)	23(50%)	11(23.9%)
6. I try to make sure that contents transmitted to students are clear and understood by them.	0(0.0%)	1(2.2%)	5(10.9%)	19(41.3%)	21(45.7%)
7. I try to listen to students’ arguments before any judgement.	0(0.0%)	0(0.0%)	10(21.7%)	21(45.7%)	15(32.6%)
8. I seek students’ feedback about the difficulties related to teaching form and content.	0(0.0%)	2(4.3%)	12(26.1%)	20(43.5%)	12(26.1%)
9. I understand the systemic process of learning and the challenges faced by the students.	0(0.0%)	0(0.0%)	11(23.9%)	25(54.3%)	10(21.7%)
10. I have appropriate communication skills and behavior as a receiver.	0(0.0%)	0(0.0%)	2(4.3%)	36(78.3%)	8(17.4%)

**Table 2:** Questionnaire about students’ perception of the quality of professors’ communications in the process of learning Dentistry.

Students’ perceptions	Strongly disagree 1	Disagree 2	Neither agree nor disagree 3	Agree 4	Strongly agree 5
1. Professors develop appropriate teaching methods for the different learning situations.	3(2.6%)	25(21.9%)	29(25.4%)	50(43.9%)	7(6.1%)
2. Professors present content in a logical and coherent context.	0(0.0%)	11(9.6%)	28(24.6%)	62(54.4%)	13(11.4%)
3. Professors have good communication skills and make clear analogies.	0(0.0%)	20(17.5%)	35(30.7%)	49(43%)	10(8.8%)
4. Professors use and competently apply new technologies as strategies in the learning process.	3(2.6%)	27(23.7%)	38(33.3%)	40(35.1%)	6(5.3%)
5. Professors are confident about the quality of communications and its impact on students’ learning.	2(1.8%)	14(12.3%)	40(35.1%)	42(36.8%)	16(14%)
6. Professors have a clear understanding of the contents transmitted to students.	0(0.0%)	9(7.9%)	8(7%)	62(54.4%)	35(30.7%)
7. Professors try to listen to students’ arguments before any judgement.	21(18.4%)	48(42.1%)	26(22.8%)	15(13.2%)	4(3.5%)
8. Professors try to give feedback to students about the difficulties related to teaching form and content.	25(21.9%)	37(32.5%)	24(21.1%)	25(21.9%)	3(2.6%)
9. Professors understand the systemic process of learning and the challenges faced by the students.	16(14%)	30(26.3%)	44(38.6%)	22(19.3%)	2(1.8%)
10. Professors have appropriate communication skills and behaviors as receivers.	10(8.8%)	14(12.3%)	43(37.7%)	36(31.6%)	11(9.6%)

## Results

The questionnaires were applied to professors (α=0.753) and students (α=0.835). Of the total number of participants that met inclusion criteria, 46 (76.6%) professors and 114 students (63,3%) answered the questionnaires. 

Tables 1 and 2 show the results. There were statistically significant differences between professors’ self-perceptions and students’ perceptions of the quality of their communications (p<0.001) (Table 3). 

**Table:** 3. Analysis of professors’ self-perceptions and students’ perceptions about the communication process.

		Scores					Student t test		
		*Mean*	*SD*	*t*	*df*	*p*	Mean difference	95% C coefficient	Effect size (Cohen’s d)
Communication	Professors	4.05	0.38	10.67	130.4	<0.001	0.86	0.70-1.02	1.69
	Students	3.19	0.61		43				

SD - Standard deviation; CI - Confidence interval; Professors n = 46 (76.6%); Students n = 114 (63.3%)

One of the specialists in the construct under study, who was sent the questionnaire for the analysis of content validity, suggested a change in the wording of one of the items in the questionnaire for professors, the one that referred to giving students feedback. This item was rewritten to make the question clearer.

Professors’ self-perceptions suggested a highly positive professional performance in sending information to students, often developing appropriate methods, with logical content, good communication skills, and confidence. Moreover, their self-perception of reception by the students was also positive. The highest values were found for often confirming content clarity and understanding and listening to students' arguments, trying to give them feedback before making any judgment, understanding the systemic learning process, behaving appropriately, and having the ability to be a good receiver in the communication process. In 78.3% of the instances of professors’ self-perceptions, an appropriate behavior was chosen as “often” for communication skills as a receiver in the process (Table 1). The frequency of this same item was classified as “often” in only 31.6% of students’ perceptions. The open questions were answered by a small number of professors, and communications were classified very heterogeneously among them. There was no analysis of the learning process, including feedback, by students. 

Students’ perceptions were significantly different (p<0.001) from the professors’ self-perceptions of their communication process. In the group of students, 47.3% (54) disagreed or neither agreed nor disagreed with professors in the application of appropriate methods for different learning contexts. In 43.9% (50) of the questionnaires, students agreed that this is an approach used by professors. In general, in the process of sending information, the predominant student perception was that they did not agree or disagree with the questionnaire statement. In 23.7% ^27^ of the students disagreed with the statement that new technologies were used and prevalent as strategies in the learning process. Contrary to what was expressed by professors, students had an unsatisfactory perception of professors in the item about reception in the communication process and their appropriate behaviors and communication skills as a receiver, which should include listening to students' arguments before making any judgment, giving feedback when teaching difficult contents in complex ways, understanding the systemic process that involves learning, and understanding the challenges that face students (Table 2). Although a small number of students answered the open question, there were indications that the difficulties in learning were associated with the barrier raised between professors and students, and the challenges that face professors to build trust in their relationship with students. Another aspect brought up by students was the need to have positive communication with professors, as they understand that this is not representative of the group of professors. 

## Discussion

Professors’ self-perception of their communication skills as a reference for sending and receiving information was satisfactory. Students did not strongly agree with the statements about the quality of their communication process, particularly the items about confirming content understanding, listening to students’ arguments, being available for feedback in case of difficulties, understanding the systemic process that involves learning, and even having good communication skills as receivers. The answers to the open question revealed the heterogeneity of the knowledge transfer performance by professors, and the need to establish feedback for a possible reinforcement moment.

Communication takes place in a context of great importance in learning, as it directly affects factors that are relevant for clarity, safety, confidence, and the logic of the contents taught. Success in a career as a professor depends on a high communicative potential. The diversity of strategies and methodological and technological teaching resources are facilitating and stimulating factors for students. Professors and students’ understanding of and commitment to the circumstances and implications of the learning process reveal the sophisticated role that each individual plays in this context. Both professors and students should assume their roles as protagonists in educational learning practices, characterized by the complexity of this process ^1^. 

The analysis of the study methods revealed that the questionnaires were structured to evaluate the quality of the self-perceptions on the communication skills between professors and students and the students’ perceptions of this process, considering their effect on the learning process. For this purpose, this study used a self-report model of closed questions in a five-point Likert scale ^23^, complemented by a question for each group. During the construction of the questionnaire, specialists in the construct under study analyzed it to evaluate the validity of its contents. 

The instruments used in this study were based on observations of the theoretical framework used in previous studies about questionnaires ^8^ ^,^ ^9^ ^,^ ^10^ ^,^ ^11^ and professors’ and students’ communicative competence. Bandrés et al. ^8^ developed a questionnaire to analyze the communicative competence of training teachers with a teaching degree. In the item about students’ perceptions of their verbal communicative competence, their assessment of their abilities as senders was poorer than those as receivers. Verbal expression was also perceived as particularly limited in more formal communicative events. Gallego-Ortega and Rodríguez-Fuentes ^11^ evaluated the perception of communicative competence in a group of 115 university students in a Primary Education course, majoring in special education. Students admitted having an insufficient mastery of this competence, with a slight improvement in communicative skills during the course. Segovia et al. ^9^ used a questionnaire to evaluate the communication skills of students and future professors in undergraduate teaching courses. Most professors believed that they mastered this competence sufficiently well (44.7%) or at a high level (22.4%) in their classes. The results of students’ communication skills as senders indicated that 43.9% considered their skills insufficient, and 40.3% believed that they were sufficiently proficient. Study results suggested that these skills should be improved during the course by including teaching strategies aimed at improving the communicative skills of future teachers. 

Communications also play an important role in other professional segments, such as in interpersonal relationships between professor and professor, professor and student, professor and patient, student and student, student and patient, and student and clerk. Therefore, communications have been a reason for the growing attention paid to the healthcare professions in recent years, particularly in what concerns education and advanced professional formation. Some studies have reported the positive effects of communication on patient satisfaction, adherence to treatment, and even treatment outcomes. Therefore, communication skills should have more room in teaching guidelines during courses that prepare for healthcare professions ^12^ ^,^ ^13^ ^,^ ^14^ ^,^ ^15^ ^,^ ^16^ ^,^ ^17^ ^,^ ^18^ ^,^ ^19^. 

Several countries have increased the standards of communicative competencies in medical education, and their medical licenses require passing tests on communicative skills as part of national professional exams ^4^ ^,^ ^5^ ^,^ ^12^ ^,^ ^13^ ^,^ ^14^ ^,^ ^15^ ^,^ ^16^ ^,^ ^17^ ^,^ ^18^ ^,^ ^19^.

Training health professionals in communication skills has been a promising approach to changing behaviors and attitudes in this area. Studies have shown that patients benefit from being seen by trained health professionals, effectively prepared to use the power of communication skills. However, scientific evidence from sound investigations about this topic is still lacking ^19^. A communications training model that has achieved important results is the application of the objective structured clinical examination (OSCE), which motivates the student to use the structured communication process with patients ^18^.

Technical skills as content in the curricular structure of a course preparing for a specialty may be an important differential factor. Some contents in Dentistry, for example, explore the communication process, that is, are studied as behavioral skills. Specific knowledge components are essential. However, those whose mission is to support the formation of qualified human capital are faced with an additional basic challenge: to understand the role and impact of communications and confidence on the quality of learning procedures and productivity. At the same time, challenging fragilities have to be overcome. This includes deficient feedback and communication noises, as well as the lack of training and development as agents of a culture of learning and innovation. Some requisites are essential to achieve project outcomes in a contemporary organization whose purpose is the formation of qualified human capital. They include communication, confidence, and the ability to propose solutions.

Therefore, student behaviors about the content and form of the information received in an undergraduate course deserve a more critical analysis from a pedagogical perspective. Santos-Neto and Franco ^24^ contextualized the pedagogical challenges faced by professors facing new generations, considering the present and the future. Of the several aspects discussed, the daring opening of professors to learning new forms of communication, especially the languages of images, computation, and multimedia, may bring them closer to younger generations and the contemporary forms of knowledge construction. Moreover, the promotion of new narratives is associated with the formation in and for the entirety, with creative syntheses and the ability to keep dialogues ^24^.

The forms of communication have increased fantastic changes within a complex universe. Form and content within a logical and efficient sense of communication have the potential to define positive relationships within a learning culture. The level of interpretation of a professor’s communication framework is processed by students and analyzed within the proper context for any knowledge that has been presented. A learning strategy used by a professor may make a difference in the potential in sharing new information, as its practical use may result in a higher level of retention and a better application of what was learned. In this sense, a reading habit is fundamental to expanding vocabulary developing logical thinking, and the ability to interpret and think assertively. A competence to reason accurately affects coherence and supports the development of the right narrative. Learning models with a practical application of knowledge have an impact on student behavior and naturally induce a proactive attitude. At the same time, it leads to greater interest, involvement, and capacity to retain information.

A rational and practical application of knowledge, in which students find the actual meaning of what they are learning and professors have the opportunity to analyze the potential of new talents, is based on an important framework ^1^ ^,^ ^2^ ^,^ ^3^ ^,^ ^4^ ^,^ ^5^ ^,^ ^8^ ^,^ ^9^ ^,^ ^10^ ^,^ ^11^ ^,^ ^12^ ^,^ ^20^ ^,^ ^21^ ^,^ ^24^ ^,^ ^25^ ^,^ ^26^ ^,^ ^27^ ^,^ ^28^. Learning management and culture should be powerfully implemented as part of the strategies of an organization that aims to achieve success ^29^.

Professional career management in Dentistry it has been highlighted as one of the determining factors of success in an individual's life. Initial reflection on career excellence professionals involves four categories of analysis, including human formation, skills acquired during undergraduate/postgraduate studies, focus and engagement professional, and, finally, the labor market reference. In all these categories, the ability to communicate clearly and competently has been an important reference ^29^. 

The future of a teaching career in Dentistry, as well as the selection of a specialty within this profession, may be affected by the behavior, communication practices, and confidence transmitted by professors. Therefore, a graduate course, either a master's or doctorate, taken by a professor should incorporate a culture of permanent learning and innovation ^29^. The relevance of adjustment in the communication process implies the establishment of behavior patterns with the capacity to understand the human perspective in the face of the challenges inherent to each generation and new learning processes. The challenges and limitations involve breaking the zones of complacency and comfort that must be incorporated as necessary changes. At the same time, technological resources require permanent updates, which has impacted the act of learning. Furthermore, communication skills are essential in the teaching career and for health professionals. Further studies should be carried out to investigate the reading habits and cultures of university teachers and students to identify weaknesses that may be risk factors and limitations in communication skills.

## Conclusion

Professors' self-perceptions and students' perceptions of the quality of communication between university teaching staff in student learning had statistically significant differences. Professors' self-perception of sending and receiving information was considered satisfactory, whereas students did not fully agree, particularly about the items about communication reception. The perceptions identified with the use of the questionnaire may lead to a new direction in the communicative behavior of professors.
